# Erector spinae plane block improves postoperative recovery after laminectomy and discectomy surgery: a retrospective cohort study

**DOI:** 10.1186/s12871-023-02271-1

**Published:** 2023-09-12

**Authors:** Renee J. C. van den Broek, Valerie M. M. van Meegen, Hazem Al Khawaja, R. Arthur Bouwman, Barbara Versyck

**Affiliations:** 1 Department of Anesthesiology, Intensive Care and Pain Medicine, Catharina Hospital, Michelangelolaan 2, 5623 EJ Eindhoven, the Netherlands; 2 Department of Epidemiology, Faculty of Health, Medicine and Life Sciences, CAPHRI School for Public Health and Primary Care, Maastricht University, Universiteitssingel 40, 6229 ER Maastricht, the Netherlands; 3 Department of Anesthesiology, Maastricht University Medical Center, P. Debyelaan 25, 6229 HX Maastricht, the Netherlands; 4 Department of Neurosurgery, Catharina Hospital, Michelangelolaan 2, 5623 EJ Eindhoven, the Netherlands; 5 Department of Neurosurgery, Elisabeth Tweesteden Hospital, Hilvarenbeekseweg 60, 5022 GC Tilburg, the Netherlands; 6 Department of Electrical Engineering, Eindhoven University of Technology, Groene Loper 19, 5612 AP Eindhoven, the Netherlands; 7 Department of Anesthesiology, AZ Turnhout, Steenweg Op Merksplas 44, 2300 Turnhout, Belgium

**Keywords:** Erector spinae plane block, Functional recovery, Laminectomy, Postoperative pain, Regional anesthesia, Spinal surgery

## Abstract

**Background:**

There is still room for improvement of pain management after spinal surgery. The goal of this study was to evaluate adding the erector spinae block to the standard analgesia regimen. Our hypothesis was that the erector spinae plane block will decrease length of hospital stay, reduce opioid need and improve numeric rating scale pain scores.

**Methods:**

This was a single center retrospective cohort study. We included 418 patients undergoing laminectomy or discectomy from January 2019 until December 2021. The erector spinae plane block was introduced in 2016 by Forero and colleagues and added to our clinical practice in October 2020. Patients who did not receive an erector spinae plane block prior to its implementation in October 2020 were used as control group. The primary outcome measure was functional recovery, measured by length of hospital stay. Secondary outcome measures were perioperative opioid consumption, need for patient-controlled analgesia and numeric rating scale pain scores. Postoperative data collection time points were: at the PACU and after 3, 6, 12 and 24 h postoperatively.

**Results:**

There was a significant shorter length of hospital stay in patients undergoing single level laminectomy (with erector spinae plane block 29 h (IQR 27–51), without block 53 h (IQR 51–55), *p* < .001), multiple level laminectomy (with erector spinae plane block 49 h (IQR 31–54), without block 54 h (IQR 52–75), *p* < .001) and discectomy (with erector spinae plane block 27 h (IQR 25–30), without block 29 h (IQR 28–49), *p* = .04).

**Conclusions:**

Erector spinae plane block reduces length of stay after laminectomy surgery.

## Background

Pain management after spinal surgery remains complicated. Preoperative opioid use, is the strongest predictive risk factor for developing poorly controlled pain in spine surgery [1]. Additionally, even in patients determined to be at low risk for poorly controlled pain, one third of all patients has poor pain control postoperatively [2]. Fourteen percent of patients with disc herniation and about one third of patients with spinal stenosis has poorly controlled pain during the first 24 h after surgery [2, 3]. These findings explain why the erector spinae plane (ESP) block is increasingly studied in patients undergoing spine surgery, as it is a relatively easy technique to learn and implement for postoperative pain control in these patients. In our own experience the ESP block reduced postoperative pain and length of stay after posterior lumbar interbody fusion surgery [4]. There is one animal study supporting this hypothesis. ESP block was associated with reduced perioperative opioid consumption, intraoperative adjuvant analgesic use and incidence of pharmacological interventions to treat cardiovascular complications in dogs undergoing a hemilaminectomy [5]. In humans, this was confirmed by a meta-analysis showing a reduction in postoperative pain scores and opioid use in patients undergoing different types of spine surgery [6]. Only one RCT on ESP block in patients undergoing lumbar disc herniation repair has been performed, which showed a reduction in postoperative morphine consumption [7]. There are no studies yet on the effect of ESP block in patients undergoing laminectomy. Also, in studies on the effect of ESP block in spine surgery, length of stay is not frequently reported.

Functional recovery is the single most important target of recovery after surgery [8]. Since discharge criteria after surgery are increasingly standardized, one can assume patients have reached an adequate and equal level of functional recovery at time of discharge [9, 10]. Therefore, the primary outcome parameter is length of hospital stay. In this study we evaluated the effect of the ESP block in patients undergoing laminectomy and discectomy. Our hypothesis is that the ESP block will decrease length of stay (LOS), reduce opioid need and improve numeric rating scale (NRS) pain scores.

## Methods

### Aim, design and setting

The goal of this study was to evaluate adding the erector spinae block to the standard analgesia regimen. This is a retrospective cohort study. Patients who underwent discectomy or laminectomy from January 2019 until December 2021 were included in this study. Since October 2020 the ESP block has been added to the anesthesia treatment. Anesthesiologists with extensive experience in regional anesthesia techniques performed the blocks. Informed consent for the ESP block was obtained at the pre-anesthesia consultation clinic. All patients who underwent discectomy or laminectomy in 2019 and 2020 before the introduction of ESP blockade were used as control group. An informed consent was obtained from all individuals included in the study for use of their data, analysis and publication.

### Surgery

All patients underwent lumbar decompressive spinal surgery; discectomy or laminectomy, in the prone position without instrumentation. In the majority of cases, this included L2-3, L3-4 and/or L4-5. Laminectomy was performed through an interlaminar decompression. Bilateral or unilateral approach was chosen depending on the side of complains. Discectomy was performed through an unilateral approach with a classical linear incision. One neurosurgeon (HK) performed the surgical procedure in all patients in this study.

### Perioperative management

This study was designed identically to our previous evaluation of ESP block in patients undergoing posterior lumbar interbody fusion surgery [4]. Briefly, all patients underwent general anesthesia. Induction was done by propofol, sufentanil and rocuronium or succinylcholine. Anesthesia was continued by sevoflurane and sufentanil. All patients received postoperative nausea and vomiting prophylaxis by dexamethasone 8 mg and ondansetron 1 mg. Pain treatment based on the patient’s pain score included acetaminophen preoperatively, sufentanil intraoperatively, morphine on the PACU and a combination of acetaminophen, NSAIDs and opioids (morphine or oxycodone) on the ward. Neuraxial blocks or other locoregional anesthesia techniques including wound infiltration were not administered. Routine assessment of numeric rating scale (NRS) scores of pain, postoperative nausea and vomiting (PONV) and complications was done at the postoperative care unit (PACU) and the ward at set intervals.

### Erector spinae plane block

**Fig. 1:**
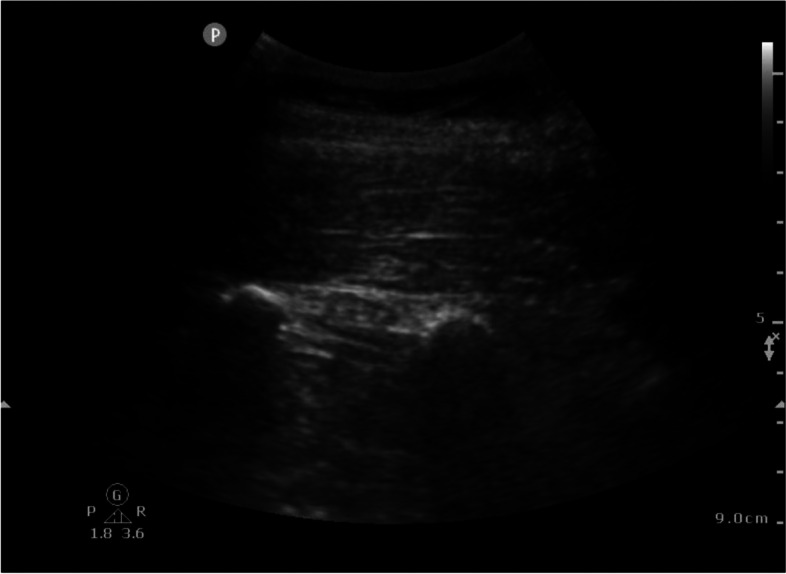
Ultrasound image during the ESP block procedure; at the top of the image the erector spinae muscles and below them the transverse processes of T12 and L1

The ESP block was performed as described by Forero et al. and as reported in our previous study on posterior lumbar interbody fusion surgery [4, 11]. We used an ultrasound machine (Philips Sparq, Amsterdam, the Netherlands) with a high-frequency curved array probe (Philips IPx-7 C5-1 PureWave, Amsterdam, The Netherlands) and a 10-cm 21 gauge ultrasound-needle (Pajunk SonoPlex STIM, Geisingen, Germany). Radiologic studies have demonstrated the spread of local anesthetics to the lumbar plexus when performed at a lumbar level [12]. Therefore we chose to perform the ESP block at T12 to decrease the chance of motor block. The ultrasound probe was placed 2–3 cm lateral to the vertebral column in longitudinal alignment to obtain the ultrasound image as seen in Fig. 1. The needle was inserted in-plane in a cephalad to caudal direction. After bone contact with the tranverse process was obtained, the needle was retracted slightly. Hydrodissection with normal saline (NaCl 0.9%) was performed to identify and open up the correct plane. After confirmation of correct placement of the needle, a dose of 20 ml of ropivacaine chloride was injected. The ESP block was performed bilaterally after induction and placing the patient in prone position but before the start of surgery. Total ropivacaine dose was 200 mg (40 ml) for patients over 70 kg, 150 mg (40 ml) for patients 50–70 kg and 3 mg kg^−1^ (40 ml) for patients under 50 kg.

### Data collection

Data was registered in the hospitals patient data management system. The collected information included preoperative data from the pre-anesthesia and surgical consultations, intra-operative registration of medication as reported by the attending anesthesiologist and postoperative documentation of the patient’s condition, including NRS scores, routinely registered by nurses on the PACU and surgical ward. Postoperative data collection time points were: at the PACU and after 3, 6, 12 and 24 h postoperatively. After assessment by the neurologist and physiotherapist, patients were discharged home if they were able to self-care, the pain was manageable and there were no new neurologic deficits.

### Statistical analysis

Functional recovery as measured by LOS was the primary outcome parameter. Opioid consumption during the first 24 h post-surgery, the need for PCA and NRS scores were secondary outcome measures. Continuous variables were presented as mean and standard deviation (SD) or median and interquartile range (IQR), depending on normality. Categorical variables were reported as a number or a percentage.

Differences in normal distributed continuous variables between groups were tested using an independent T-test. Differences in not-normal distributed continuous variables between groups were tested using a Mann–Whitney U-test. Differences in categorical variables between groups were tested using a Fisher’s exact test. A *p*-value < 0.05 was considered statistically significant. All statistical analyses were performed in collaboration with the research department under guidance of Saskia Houterman, statistician.

### Ethical considerations

The Medical Research Ethics Committees United (MEC-U, Nieuwegein, the Netherlands) (document number W21.024 and AW22.025) approved this study. It was executed according to the Declaration of Helsinki (Fortaleza, Brazil, October 2013), the Medical Research Involving Human Subjects Act (WMO) and Good Clinical Practice guidelines. To report this study, we followed the STROBE (STrengthening the Reporting of OBservational studies in Epidemiology) guidelines.

## Results

In total 418 patients were included in the study; 223 patients underwent laminectomy of 1 level, 111 patients underwent laminectomy of multiple levels, 84 patients underwent discectomy (Table 1). In the single level laminectomy group 33% of patients received an ESP block, in the multilevel laminectomy group 28% and in the discectomy group 26%. For the three groups, sex and ASA classification were equally distributed between the patients who received an ESP block and who did not. Age was equally distributed in the group of patients who underwent multilevel laminectomy and discectomy. On average, patients who underwent single level laminectomy were 5 years younger in the ESP group. Preoperative opioid and SSRI use was equal in the discectomy and multiple level laminectomy group. In the single level laminectomy group, patients who received an ESP block used SSRI’s and the combination of opioids and SSRIs more often. On average, duration of surgery was 8 min longer for patients who underwent single level laminectomy and 9 min longer for discectomy patients who received an ESP block. For the group who underwent multilevel laminectomy, duration of surgery was similar for patients who did and who did not receive an ESP block.

**Table 1:** Patient characteristics

	**Laminectomy 1 level (*n* = 223)**			**Laminectomy multilevel (*n* = 111)**			**Discectomy (*n* = 84)**		
	**ESB yes (*n* = 73)**	**ESB no (*n*-150)**	***p*- value**	**ESB yes (*n* = 31)**	**ESB no (*n* = 80)**	***p*-value**	**ESB yes (*n* = 22)**	**ESB no (*n* = 62)**	***p*-value**
**Male sex, *n* (%)**	37 (51)	82 (55)	.668	20 (65)	39 (49)	.145	9 (41)	25 (40)	1.000
**Mean age (IQR) in years**	57 (37–77)	62 (43–81)	.009	69 (55–83)	68 (56–80)	.860	44 (23–65)	43 (28–58)	.657
**ASA 1, *n* (%)**	7 (8)	11 (8)	.581		2 (3)	.082	6 (29)	19 (31)	.282
**ASA 2, *n* (%)**	48 (68)	93 (63)		12 (39)	46 (58)		14 (68)	37 (61)	
**ASA 3, *n* (%)**	16 (23)	41 (28)		18 (58)	31 (40)		1 (5)	5 (8)	
**ASA 4, *n* (%)**		2 (1)		1 (3)					
**Mean length of surgery ± SD in hrs**	71 ± 10	63 ± 12	< .001	87 ± 16	85 ± 19	.569	71 ± 12	62 ± 11	.003

An independent samples T-test was used to compare means; for categorical variables a Fisher’s exact test was used

*SD* Standard deviation, *ASA* American Society of Anesthesiologists

### Single level laminectomy

Patients with an ESP block had a significant shorter LOS (with ESP 29 h (IQR 27–51), without ESP block 53 h (IQR 51–55), *p* < 0.001). Opioids during surgery, on the PACU, oral opioids on the ward and the amount of patients needing a PCA pump were similar for both groups, as were NRS scores at all time-points (Table 2).

**Table 2:** Length of stay, opioid use and NRS scores

	**Laminectomy 1 level (*n* = 223)**			**Laminectomy multilevel (*n* = 111)**			**Discectomy (*n* = 84)**		
	**ESB yes (*n* = 73)**	**ESB no (*n*-150)**	***p*- value**	**ESB yes (*n* = 31)**	**ESB no (n = 80)**	***p*-value**	**ESB yes (*n* = 22)**	**ESB no (*n* = 62)**	***p*-value**
**Median of LOS (IQR) in hrs**	29 (27–50)	53 (51–55)	< .001	49 (31–54)	54 (52–75)	< .001	27 (25–30)	29 (28–49)	.04
**Mean of sufentanil ± SD (mcg)**	19 ± 6	19 ± 5	.401	20 ± 5	20 ± 5	.998	17 ± 5	19 ± 5	.127
**Mean of morphine at PACU ± SD (mg)**	9 ± 5	9 ± 4	.740	11 ± 6	10 ± 5	.510	8 ± 5	9 ± 3	.399
**Mean of OME OR/PACU ± SD**	53 ± 20	52 ± 22	.585						
**Median of OME OR/PACU (IQR)**				50 (40–70)	55 (40–70)	.572	50 (39–62)	52 (50–64)	.715
**PCA morphine (yes) *n*, (%)**	3 (4)	7 (5)	1.000	2 (7)	4 (5)	.671	1 (5)	5 (8)	1.000
**Median of oxycodone 48 h (IQR) in mg**	10 (5–30)	10 (5–20)	.401	10 (5–15)	15 (5–20)	.201	10 (3–13)	13 (6–20)	.186
**Mean NRS start PACU ± SD**	2.0 ± 1.8	2.2 ± 2.4	.567	1.3 ± 1.8	2.2 ± 2.2	.106	2.6 ± 2.4	2.5 ± 2.3	.871
**Mean NRS end PACU ± SD**	2.5 ± 1.9	2.9 ± 2.2	.277	2.4 ± 1.8	2.8 ± 2.3	.451			
**Median NRS end PACU (IQR)**							3 (2–3)	3(2–3)	.432
**Mean NRS 3 h ± SD**	3.6 ± 2.1	3.3 ± 2.2	.455	3.4 ± 2.1	3.4 ± 2.2	.921	3.4 ± 1.6	3.8 ± 2.3	.567
**Mean NRS 6 h ± SD**	3.6 ± 2	3.7 ± 2.2	.802	2.2 ± 1.8	3.2 ± 2.5	.219	2.3 ± 1.2	4.0 ± 2.2	.031
**Mean NRS 12 h ± SD**	3.5 ± 2.0	3.9 ± 2.5	.264	2.9 ± 2.2	3.7 ± 2.2	.156	4.8 ± 2.4	3.6 ± 2.4	.065
**Mean NRS 24 h ± SD**	3.8 ± 2.4	3.5 ± 2.4	.527	2.8 ± 1.9	3.6 ± 2.5	.132	5.7 ± 2.0	4.2 ± 2.3	.141

An independent samples T-test or Mann- Whitney U-test was used to compare means; for categorical variables a Fisher’s exact test was used

*LOS* Length of Stay, *IQR* Inter Quartile Range, *PCA* Patient Controlled Analgesia, *NRS* Numeric Rating Scale

### Multilevel laminectomy

Patients with an ESP block had a shorter LOS (with ESP 49 h (IQR 31–54), without ESP block 54 h (IQR 52–75), *p* < 0.001). Opioids during surgery, on the PACU, oral opioids on the ward and the amount of patients needing a PCA pump were similar for both groups, as were NRS scores at all time-points (Table 2).

### Discectomy

Patients with an ESP block had a shorter LOS (with ESP 27 h (IQR 25–30), without ESP block 29 h (IQR 28–49), *p* = 0.04. There was no difference in opioids during surgery, on the PACU, oral opioids on the ward and the amount of patients needing a PCA pump between patients with and without an ESP block (Table 2). NRS scores at the start and end of PACU stay were similar for both groups. NRS scores 6 h postoperatively were lower in the group with ESB (2.3 ± 1.2) than without ESP block (4.0 ± 2.2, *p* = 0.031). NRS scores at 3, 12 and 24 h postoperatively were similar for both groups (Table 2).

Because of the retrospective character of the study, the amount of morphine used by patients who had a PCA pump was not registered. Therefore a relevant comparison of opioid consumption between the PCA and oxycodone group was not possible. Complications related to the ESP block did not occur. Subgroup analysis for the different levels of surgery was not performed because most subgroups were too small to draw relevant conclusions.

## Discussion

**Fig. 2:**
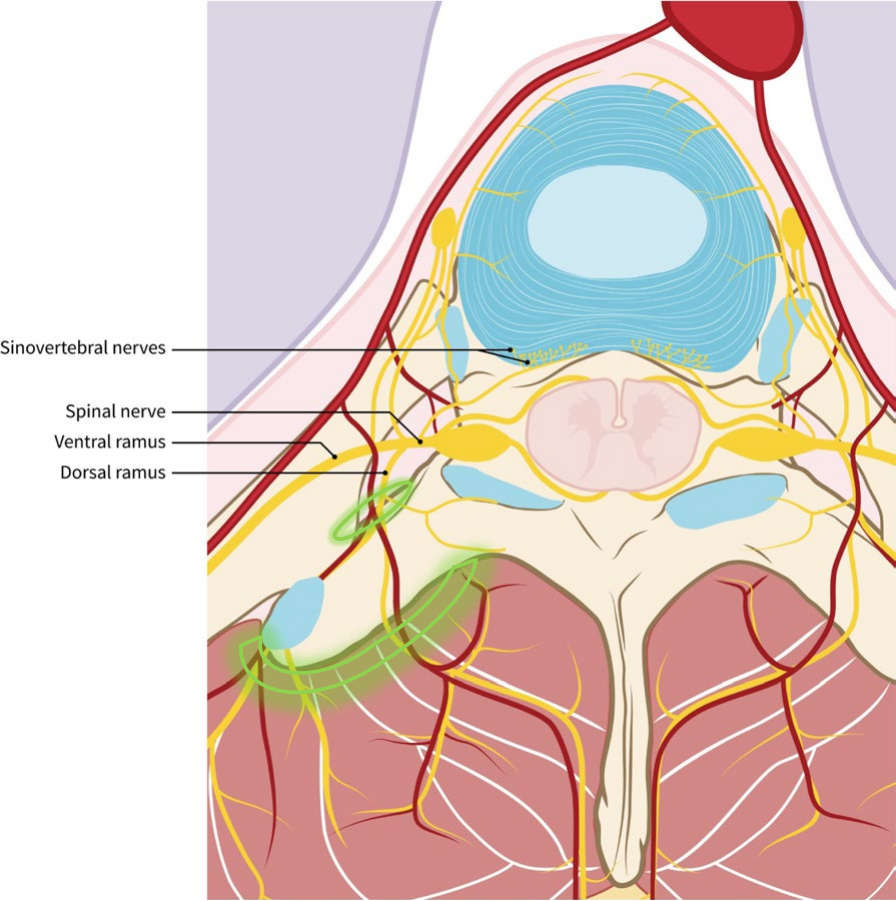
Sensory innervation of the vertebra, discus and surrounding muscles. Green area: spread of local anesthetic

This study evaluated the introduction of the ESP block to standard anesthetic care in 418 patients undergoing single and multilevel laminectomy and discectomy. ESP block reduces LOS significantly in all three groups, with the most significant reduction observed in the single level laminectomy group. We hypothesize that the reduction in LOS is due to decreased postoperative lower back pain resulting from intraoperative manipulation of the back muscles. Patients who wake up without soreness or stiffness are able to begin mobilizing sooner, ultimately leading to a shorter length of stay [13, 14]. As patients are stimulated to mobilize as soon as their pain scores allow this, mobilization occurs sooner whilst pain scores remain the same. The effect of ESP block on LOS in the discectomy group was the smallest. On the one hand sensory innervation of the intervertebral disc is mediated by the sinuvertebral nerves, which are formed by somatic roots from the ventral ramus and by autonomic roots from the gray ramus communicans (Fig. 2) [15]. Since the ESP block only consistently blocks the dorsal branches of the spinal nerves, it makes sense that there is less effect in the discectomy group [16, 17]. On the other hand, the LOS for discectomy is already relatively short, which means that the impact of pain control on LOS is likely smaller in this procedure than in laminectomy. The reduction in LOS in single level laminectomy of 24 h is obviously clinically relevant. One can discuss the clinical meaning of the reduction of 5 h in multilevel laminectomy and 2 h in discectomy. However, with current staffing shortage on the ward, every reduction in length of stay is clinically relevant.

For patients undergoing discectomy, the ESP block also lowers the NRS score at 6 h postoperatively. This reduction of NRS 4 to NRS 2.3 lowers pain scores below what is considered bearable pain (≤ 3) and therefore seems clinically relevant although we did not measure a reduction in opioid need. In this study, the ESP had no effect on pain scores in the laminectomy groups and no effect on postoperative opioid consumption in all three groups. This is in contrast to three RCTs aimed at patients undergoing lumbar decompression surgery and laminectomy or surgery for prolapsed intervertebral disc [7, 18, 19]. These RCTs reported reduced postoperative pain scores and reduced opioid use. Possibly achieving earlier mobilization reduces the effect of ESP block on NRS scores and opioid consumption.

The reduction in length of stay could imply a significant reduction in hospital costs. An occupied hospital bed at a neurology department in the Netherlands has a cost price (including overhead) of approximately €30 per hour (source Performation benchmark, 2019). So for single level laminectomy, this is a reduction in hospital costs of €720 per patient and for multilevel laminectomy €150 per patient. In the Netherlands, approximately 6000 laminectomies of 1 level and 4000 laminectomies of multiple levels are performed per year by neurosurgeons (www.opendisdata.nl, 2019). Implementing this regional anesthesia technique can save yearly up to € 5,000,000 in medical costs.

Discectomy and laminectomy can also be done under spinal anesthesia or general anesthesia with an intrathecal injection of morphine by the surgeon at the end of surgery. Intrathecal opioids have been demonstrated to provide excellent pain relief in patients undergoing lumbar laminectomy [20, 21]. However, in the PROSPECT guideline on postoperative pain after laminectomy by the ESRA the side effects of intrathecal opioids are called worrisome, particularly because this procedure is increasingly being performed as an outpatient procedure. “These potential side effects include—but are not limited to—respiratory depression, cardiovascular stress, cognitive dysfunction, delayed wound healing, urinary and gastrointestinal dysfunction, as well as the risk of acquired tolerance and long-term opioid use. Therefore, it is prudent to avoid intrathecal opioids” [22]. There are no studies yet comparing spinal anesthesia to general anesthesia plus ESP block.

This study contains several limitations. Firstly, this is a retrospective clinical evaluation. Therefore there was no power calculation to determine the sample size which limits the generalizability of our conclusions. Secondly, staff followed our in-house protocol which allowed a more liberal anesthesia regimen and resulted in less detailed data registration compared with a strict prospective study protocol. Thirdly, the levels of operation were not homogeneous. As blocks were carried out at the T12 level in all patients and sensory block levels were not measured, there could potentially be varying degrees of block effectiveness. Fourthly, there is a lack of differentiation between pain at rest and pain during movement. This could potentially explain the absent effects on pain scores in this cohort. If individuals with an ESB tend to initiate mobilization earlier, this could potentially result in the production of pain scores comparable to those observed in the resting control group. Fifthly, the possibility of bias and confounding are inherent to a retrospective study design and may impact the ability to draw conclusions. To the best of our knowledge, during the study period the surgical techniques and treatment did not change, and there were no medical or non-medical factors influencing discharge that could potentially introduce confounding variables, such as administrative problems, social challenges, or fluctuations in staffing levels. As this is the initial study assessing the efficacy of ESP block in neurosurgical spine surgery, it highlights the direction for future research, despite the possibility of biases or confounding factors. The results provide a starting point for further determination of the role of ESP block in this field.

## Conclusions

Implementing the ESP block for laminectomy and discectomy as standard care in our center has caused a significant reduction in length of hospital stay, especially for single level laminectomy, most likely by allowing earlier mobilization.

## Data Availability

All of the individual participant data collected during the trial, after de-identification including data dictionary defining each field in the set, will be made available to investigators whose proposed use of the data has been approved by an independent review committee. This data will be available immediately following publication without any end date. Proposals should be directed to renee.vd.broek@catharinaziekenhuis.nl with a signed data access agreement.

## Abbreviations

ESP: Erector spinae plane
LOS: Length of stay
NRS: Numeric rating scale
ASA: American society of Anesthesiologists
PACU: Post anesthesia care unit
PCA: Patient controlled analgesia
SD: Standard deviation
IQR: Inter quartile range
